# Photoplethysmogram Recording Length: Defining Minimal Length Requirement from Dynamical Characteristics

**DOI:** 10.3390/s22145154

**Published:** 2022-07-09

**Authors:** Nina Sviridova, Tiejun Zhao, Akimasa Nakano, Tohru Ikeguchi

**Affiliations:** 1 Department of Information and Computer Technology, Faculty of Engineering, Tokyo University of Science, 6-3-1 Niijuku, Katsushika, Tokyo 125-8585, Japan; tohru@rs.tus.ac.jp; 2 International Research Center for Neurointelligence, The University of Tokyo, 7-3-1 Hongo Bunkyo-ku, Tokyo 113-0033, Japan; 3 Faculty of Agro-Food Science, Niigata Agro-Food University, 2416 Hiranedai, Tainai 959-2702, Japan; tiejun-zhao@nafu.ac.jp; 4 Innovation Management Organization, Chiba University, Kashiwano-ha Campus 6-2-1, Kashiwano-ha, Kashiwa-shi 277-0882, Japan; anakano@chiba-u.jp

**Keywords:** photoplethysmogram, nonlinear dynamics, nonlinear time series analysis, data length assessment

## Abstract

Photoplethysmography is a widely used technique to noninvasively assess heart rate, blood pressure, and oxygen saturation. This technique has considerable potential for further applications—for example, in the field of physiological and mental health monitoring. However, advanced applications of photoplethysmography have been hampered by the lack of accurate and reliable methods to analyze the characteristics of the complex nonlinear dynamics of photoplethysmograms. Methods of nonlinear time series analysis may be used to estimate the dynamical characteristics of the photoplethysmogram, but they are highly influenced by the length of the time series, which is often limited in practical photoplethysmography applications. The aim of this study was to evaluate the error in the estimation of the dynamical characteristics of the photoplethysmogram associated with the limited length of the time series. The dynamical properties were evaluated using recurrence quantification analysis, and the estimation error was computed as a function of the length of the time series. Results demonstrated that properties such as determinism and entropy can be estimated with an error lower than 1% even for short photoplethysmogram recordings. Additionally, the lower limit for the time series length to estimate the average prediction time was computed.

## 1. Introduction

Cardiovascular diseases (CVD), such as heart failure, stroke, and hypertension are the leading cause of death worldwide [1]. It is recognized that accessible health monitoring and early detection of CVD can be helpful in preventing and monitoring CVD. According to the World Health Organization (WHO), over three quarters of CVD cases worldwide occur in low- and middle-income countries [1], supporting the need for accessible, affordable health monitoring systems. The photoplethysmogram (PPG) is a biological signal that has been used for decades for health monitoring in clinical settings as well as in wearable devices. Besides its main applications, which are the estimation of heart rate, respiration rate, blood pressure, and oxygen saturation, PPGs are also used for vascular assessment, arterial disease and state evaluation, sleep disorders studies, and other applications [2,3,4,5]. Moreover, a number of studies reported that the PPG is also applicable for mental health monitoring [6,7,8,9,10,11]. Thus, PPG can be used for mental stress identification [6,7,8] and early detection of depression [10], which is one of the mental disorders with various social and health consequences recognized by the WHO as a leading cause of disability worldwide [12]; it is also applicable for estimation of psychiatric patients’ recovery [9]. Additionally, PPG was previously applied for occupational physiological health monitoring [13,14,15]. In addition to its wide applicability for health monitoring purposes, PPG technology is also simple and inexpensive, and—as such—it has considerable potential for use in accessible, affordable mobile health monitoring for prevention and early detection of disease, including CVD and mental disorders.

Common PPG applications require basic signal processing and analysis, such as contour analysis and time–frequency techniques [3,4,16,17,18]. Recent studies also employ neural networks and deep learning to improve the assessment or prediction of physiological states and parameters [19,20,21]. However, as the cardiac and respiratory dynamics in general [22,23,24] and the PPG dynamics in particular are recognized as deterministic chaos [9,25,26], alternative complex approaches may be required for extracting accurate information on the physiological and mental health state, as follows from previous studies [9,27]. For example, nonlinear time series analysis of PPG dynamics was applied in previous studies related to mental health monitoring [9,10,11].

There is considerable potential for extracting information about disease and health status from the PPG dynamics by using advanced analysis methods. However, the applicability of such analysis to the PPG might be limited due to the typically high measurement noise and the presence of movement artifacts [24,25,26]. The measurement noise can be limited in clinical settings, as the measurement process and settings can be strictly controlled. However, in case of measurements taken using wearable devices, both measurement noise and movement artifacts can severely hamper the extraction of information from the PPG. A common approach to address these issues involves noise filtering and motion artifact reduction, which are typically included in modern PPG devices and enable accurate estimation of heart rate, arterial oxygen saturation, etc. [28,29,30,31,32]. However, while filtering yields improvement in the estimation of these common physiological parameters, it may alter the PPG signal and affect its nonlinear dynamic features [33,34]. However, the majority of studies on PPG filtration and movement artifact reduction do not take the preservation of complex dynamics into consideration. As such, filtered signals can be efficiently used for traditional PPG applications, but they are not suitable for advanced analysis—for example, nonlinear time series analysis. As an alternate approach to limit the impact of noise and movement artifacts, it is possible to use only high-quality short segments of the recorded signal, as was done in [35]. Moreover, other advantages of using short signal segments include a reduction in computational cost, the potential for a decrease in battery consumption, and enabling real-time signal processing, all of which are particularly important in wearable device applications. However, short signal length may limit the applicability of nonlinear analysis and the accuracy of dynamic features estimates.

In studies involving the investigation of PPG dynamics, applied data length greatly varies, such as 2.1 s [36], 100 s [9], 2 min [37], 3 min or longer [10], and 5 min [25,26]. To the best of our knowledge, the applicability of nonlinear analysis methods to short PPG recordings has not been systematically investigated. Among the different methods for nonlinear time series analysis, recurrence plots, which visualize the signal dynamics as a two-dimensional binary image, and the related quantification analysis can be used to estimate the dynamical properties of time series [38,39]. Recurrence quantification analysis (RQA) was applied to the PPG to assess the effect of filtering on PPG dynamics [33] as well as in a data-driven study on hypertension from short PPG recordings [36]. However, in the latter study, the effect of the short recordings on nonlinear analysis was not discussed. Previous studies suggested that, in contrast to the majority of nonlinear time series analysis methods, RQA applied to recurrence plots is not affected by the length and drift of the data [40]. However, at the same time, for the RQA, it is generally known that longer time series provide more precise estimates of the system’s dynamical properties [39,40], as convergence of the RQA indexes is observed for sufficiently long time series. Overall, based on various reports [40], it appears that depending on the data under investigation, meaningful results might be achieved even when the time series length is recognized as short, allowing us to surmise that properties of the short-recorded PPGs might be extracted by RQA. Overall, no study so far has investigated the minimum length of a time series that can lead to reliable estimates of dynamical properties. Stated differently, the error associated with the estimates of the dynamical properties for short PPG signals is unknown.

The aim of this paper is to assess the applicability of RQA to short PPG signals obtained from healthy human subjects in reference environment, to investigate RQA indexes that can be used to extract dynamical property information from short PPG signals, and to estimate the minimum length of PPG time series necessary to keep the error below an acceptable limit. As a result, it is found that by using RQA, minimal time series length required for accurate dynamical property estimation can be elucidated, thus providing a novel technique for designing data acquisition on one hand and assuring that RQA can be applied for robust estimation of the determinism and complexity for short PPG recordings on the other.

## 2. Data

### 2.1. Photoplethysmogram

**Figure 1:**
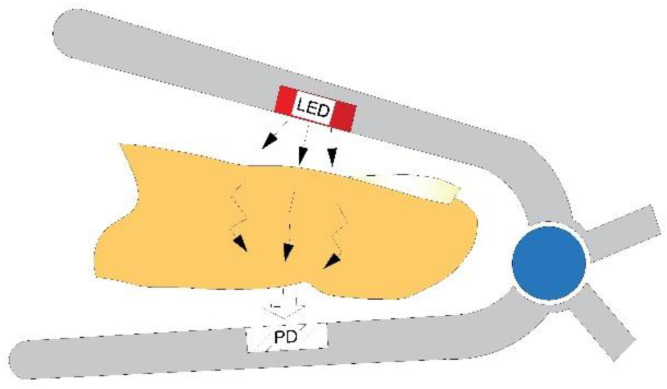
Conventional transmission PPG device setup.

Since PPG was introduced in 1937 [41], it has been widely used for heart rate monitoring, and since then, the number of its applications has expanded [2,4,18]. PPG measurement technology mainly relies on measurement by the photodetector of LED light reflected or transmitted through the skin tissue. Green, red, and near-infrared (NIR) light are the most commonly used light sources for PPG measurement. There are two main device setups depending on whether the light is transmitted or reflected. PPG device with transmission-type setup schematically shown in Figure 1 utilizes red and NIR light and is common in hospital use, while reflection-type devices utilize green light sources and are mostly applied in wearables due to the lower movement sensitivity of the green light PPGs and their flexibility in measurement location [42,43].

### 2.2. Experimental Data

In this study, the widely used NIR PPG recordings were investigated. The measurements were conducted at the Institute of Vegetable and Floriculture Science (NIVFS), NARO in 2017–2018. The experiment protocol was approved by the NIVFS ethical committee, and all participants provided informed consent prior to the experiment. Data were collected from 21–49-year-old participants in a relaxed sitting position inside an air-conditioned room with a temperature of 25 °C. Measurements were conducted for 5 min and repeated twice using a IWS920 (I.W. Technology Firm, Inc. Tokyo Devices) PPG recording device with a sampling rate 409.6 Hz.

**Figure 2:**
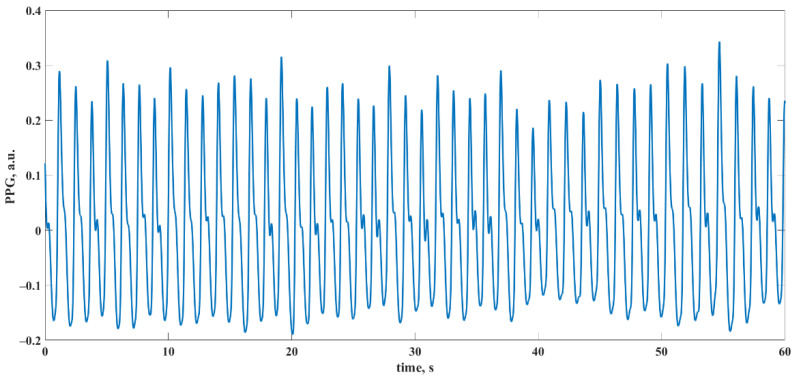
Example of a 60 s segment from a PPG recording.

Participants were asked to evaluate their temperature and comfort perception based on category scales for comfort and temperature sensation proposed in [44]. Before the experiment, participants were asked to answer a questionnaire regarding personal history of CVDs, gender, age, presence of sickness at the time of data collection, and lifestyle habits such as smoking and sports activity. Only data from nonsmoking participants with no history of CVDs and in good health condition who reported comfortable environmental conditions were included in this study. As a result, a total of 30 datasets were used. An example of a 60 s segment from one of the PPG recordings is shown in Figure 2.

Additionally, in many practical cases, lower-sampling-rate PPG data are used [45,46,47]. To broaden the applicability of this study, collected PPG recordings were two- and four-times subsampled to generate sparse PPG time series.

### 2.3. Simulated Data

**Figure 3:**
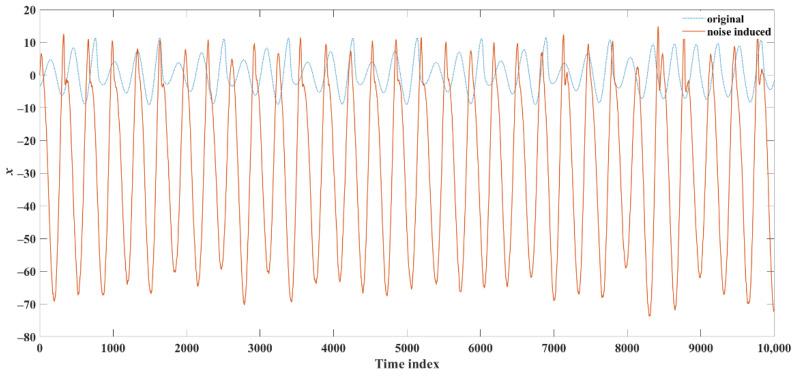
Examples of a segment of the original (blue dotted line) and noisy (red solid line) *x* time series generated using the chaotic Rössler model.

In the previous study [25] it was reported that the PPG dynamical properties show certain similarities with the noise-induced chaotic Rössler model, such as single-band-like reconstructed trajectory structure and predictability decay. Therefore, as a preliminary step, before evaluating the RQA results as a function of the length of the PPG segment, we assess the relationship between RQA results and segment length using the well-known chaotic Rössler model, described as follows:

$$\{\begin{array}{c} \dot{x}=-y-z,\\ \dot{y}=x+ay,\\ \dot{z}=b+z\left(x-c\right), \end{array}$$

where *a* = 0.2, *b* = 0.2, and *c* = 5.7 [48]. The Rössler model was numerically solved using the fourth-order Runge–Kutta method with sampling rate of 1000 Hz. To imitate the measurement noise, additive dynamical noise was introduced. On each iteration *i* of the numerical solution method, noise was added to the solution *x_i_* using the following equation: ${\tilde{x}}_{i}=x_{i}+\theta \gamma_{i}$, where θ is the noise scaling coefficient, which varies from 0 (i.e., no noise) to 0.5 in increments of 0.05; $\gamma_{i}$ is the *i*th component of a fixed uniform random noise vector; and ${\tilde{x}}_{i}$ is the input of the next iteration. For computational efficiency, the resulting time series *x* was subsampled using a factor of five. Examples of the resulting time series obtained using the original (θ = 0) and noisy (θ = 0.25) *x* time series are shown in Figure 3, where the addition of dynamical noise affected the amplitude of the time series and created additional fluctuations compared to the original time series.

## 3. Analysis

### 3.1. Time Delay Reconstruction

The time delay embedding method [49,50] is used in this study to enable nonlinear analysis, as information on the trajectory dynamics in a phase space is needed. If a variable time series ${\left\{x_{i}\right\}}_{i=1}^{n}$ consisting of *n* observations is obtained, then the points of the time-delay-reconstructed trajectory in an *m*-dimensional phase space can be calculated as in [24]:

$$X_{j}=\left(x_{j},x_{j+\tau },x_{j+2\tau },\ldots ,x_{j+\left(m-1\right)\tau }\right),$$

where *j* = 1, …, *N*, *N* = *n* − (*m* − 1)*τ* and *τ* is a time lag. In this study, the reconstruction dimension for the NIR PPG signal was set as *m* = 4 following [25], where the minimum embedding dimension was estimated by the false nearest neighbors method [51], and the time lag was defined as the time when the signal autocorrelation falls below 1/*e* [52]. It is of note that there are various approaches to the choice of the time lag, and other types of PPG signals may require adjustment of its value.

### 3.2. Recurrence Plot

The recurrence plot (RP) visualizes the dynamics of the system as a two-dimensional binary image and is calculated using the following expression:

$$R_{i,j}\left(\varepsilon \right)=\{\begin{array}{c} 0,\;if\;\|X_{i}-X_{j}\|>\varepsilon \\ 1,\;if\;\|X_{i}-X_{j}\|<\varepsilon \end{array}$$

where *i*, *j* = 1, …, *N* and *ε* is the threshold that defines the area where neighboring points of the time-delay-reconstructed trajectory are searched. If the point *X_i_* is located within the sphere centered in *X_j_* with radius *ε*, then the (*i*, *j*)-pixel is included in the RP. In this study, *ε* was equal to 10% of the reconstructed attractor size [40] defined by the maximum distance between attractor points: $\underset{i,j=1,\;\ldots ,\;N}{\max}\;\|X_{i}-X_{j}\|$.

### 3.3. Recurrence Quantification Analysis (RQA)

The RP visualizes the dynamical system as a two-dimensional image, and the displayed patterns depend on the system’s dynamics. However, RP represents a qualitative description of the system’s dynamics. RQA [39] can be applied to extract, based on the statistics of the RP, quantitative features to assess the dynamical system. In this study, the following features were estimated:

**Determinism.** Determinism (*DET*) is one of the most important properties of a dynamical system and defines whether the process can be expressed in the form of a system of equations. Determinism can be estimated as:

$$DET=\frac{\sum_{l=l_{min}}^{N}lP\left(\varepsilon ,l\right)}{\sum_{l=1}^{N}lP\left(\varepsilon ,l\right)},$$

where P(ε,l) is a histogram of diagonal lines of length *l* defined as

$$P\left(\varepsilon ,l\right)=\overset{N}{\underset{i,j=1}{\sum }}\left(1-R_{i-1,j-1}\left(\varepsilon \right)\right)\left(1-R_{i+1,j+1}\left(\varepsilon \right)\right)\overset{l-1}{\underset{k=0}{\prod }}R_{i+k,j+k}\left(\varepsilon \right),$$

and *l_min_* is a given minimum length. Ideally, for deterministic time series, *DET* is equal to unity, whereas it is lower than one when a limited number of samples are available, when the signal includes noise, etc. Practically, values of *DET* > 0.9 can be considered as a sign of determinism [53].

**Trajectory divergence.** In RQA, exponential trajectory divergence, which is an important measure of the chaotic time series, can be defined as the inverse of the length of the longest diagonal line ($L_{max}$) in the RP, expressed as:

$$L_{max}=\max\left({\left\{l_{i}\right\}}_{i=1}^{N_{l}}\right),$$

where $N_{l}=\underset{l\geq l_{min}}{\sum }P\left(\varepsilon ,l\right)$ is the total number of diagonal lines in the RP.

**Predictability.** Short-term predictability—that, is the possibility to predict future states of the signal based on past observations for a short time window—is an extremely important property both theoretically and practically. The mean prediction time of the dynamical system can be estimated by computing the average diagonal line length (*L*), defined as

$$L=\frac{\sum_{l=l_{min}}^{N}lP\left(\varepsilon ,l\right)}{\sum_{l=l_{min}}^{N}P\left(\varepsilon ,l\right)}.$$

**Complexity.** Entropy (*ENTR*) is frequently used in applied studies of RQA. *ENTR* estimates the complexity of the RP with respect to the diagonal lines and can be calculated as:

$$ENTR=-\overset{N}{\underset{l=l_{min}}{\sum }}p\left(l\right)\ln p\left(l\right),$$

where $p\left(l\right)=P\left(\varepsilon ,l\right)/N_{l}$ is the estimated probability to find a diagonal line of length *l* in the RP, and $N_{l}$ is the number of diagonal lines.

### 3.4. Error Estimation

In this study, the lower limit of the length of the time series was determined by considering the average time required for the original time-delay-reconstructed trajectory to complete one turn on the time-delay-reconstructed attractor, hereinafter referred to as “average cycle”. Specifically, the lower limit of the length of the time series was set as the time required to complete 3–5 average cycles to ensure the presence of at least one separated cycle fragment of trajectory in the neighborhood depending on the density of the reconstructed attractor trajectories. The length of the time series (*T*) assumed to lead to accurate estimates of the RQA features defined in Section 3.3 was defined as over 100 average turns of the trajectory on the reconstructed attractor. A set of RPs were computed, and RQA was performed for varying lengths of the time series. For each RQA feature *S*, the relative error $E_{l}$ associated with the estimate of *S* using a time series of length *l* with respect to the accurate measure obtained using a time series of length *T* was calculated as follows [54]:

$$E_{l}=\frac{\left|S_{l}-S_{T}\right|}{S_{T}}\times 100\%.$$

## 4. Results

### 4.1. Rössler System

**Figure 4:**
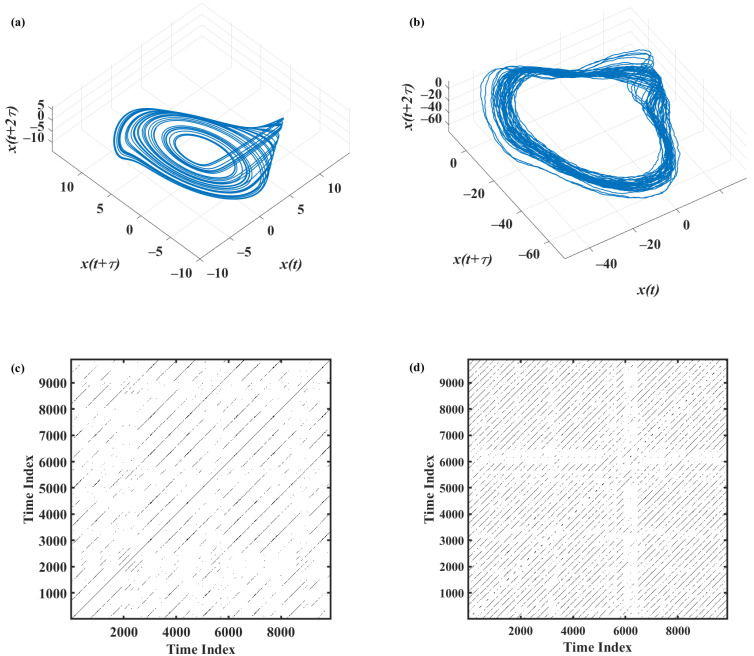
Reconstructed attractors from *x* time series of (**a**) original (*θ* = 0) and (**b**) noisy (*θ* = 0.25) Rössler systems and the resulting RPs (panels (**c**) and (**d**), respectively).

First, for the time series *x* of the original and noisy Rössler system defined in Section 2.3, the time-delay-reconstructed attractor was obtained. Figure 4a,b shows the obtained attractors for the original and noisy time series, respectively. Then, the set of RPs was calculated for varying length of the segments of the obtained trajectory of the attractor. The length of the time series varied from 1500 data points (five average cycles) to 50,000 data points (168 average cycles). For each length, 100 different segments were chosen on the attractor, and corresponding RPs were calculated. Figure 4c,d shows the RPs corresponding to a length of 10,000 points for the original and noisy trajectories shown in Figure 4a,b, respectively.

**Figure 5:**
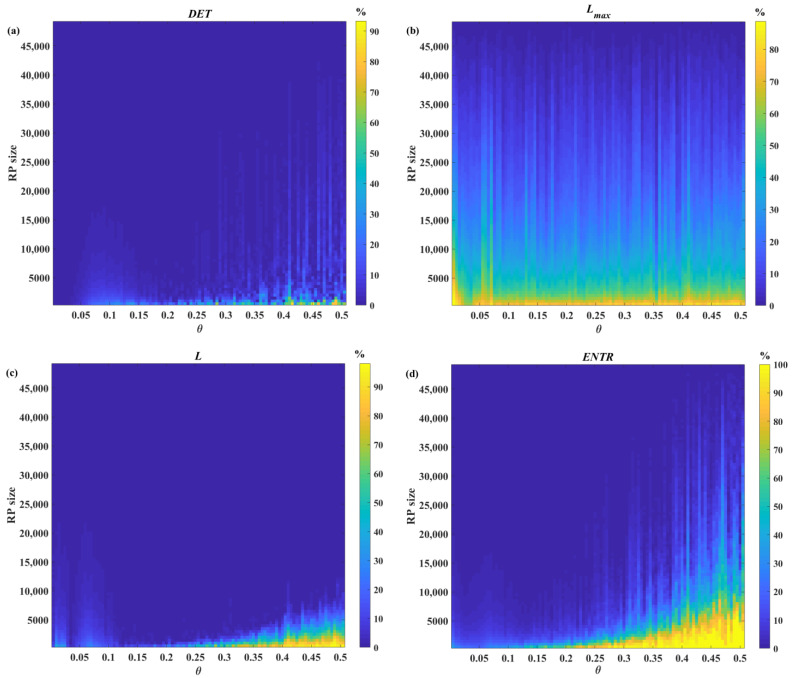
Relative error of the RQA features estimates as a function of length and noise level of Rössler time series: (**a**) determinism, (**b**) maximal diagonal line length, (**c**) average diagonal line length, and (**d**) entropy.

**Figure 6:**
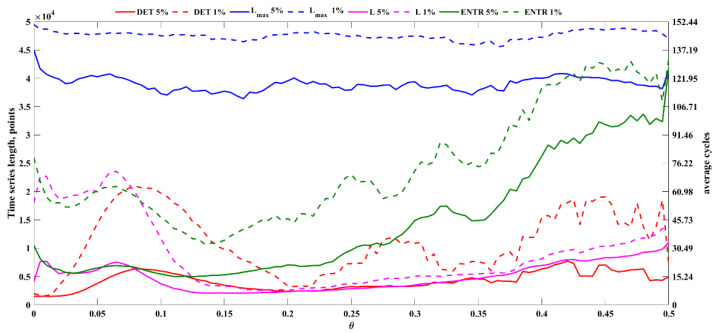
Length of the Rössler time series (left: in points, right: in average cycles) as a function of the noise level corresponding to a relative error equal to 5% (solid line) and 1% (dashed line) in the estimation of the determinism (red), maximal diagonal line length (blue), average diagonal line length (magenta), and entropy (green).

Figure 5 shows the relative errors of *DET* (a), *L_max_* (b), *L* (c), and *ENTR* (d) as a function of noise level (*θ*) and time series length, as averaged over 100 segments. Table 1 shows four calculated RQA measures reference values for three levels of noise. Table 2 shows the time series length corresponding to relative errors equal to 5% and 1% for three different noise levels. A relative error lower than or equal to 1% is considered acceptable. Therefore, the corresponding length can be used as a benchmark for the minimum length of the time series. Figure 6 shows detailed information of the length of the time series associated to relative error below 5% and 1% for the four RQA features here computed.

### 4.2. Photoplethysmogram

**Figure 7:**
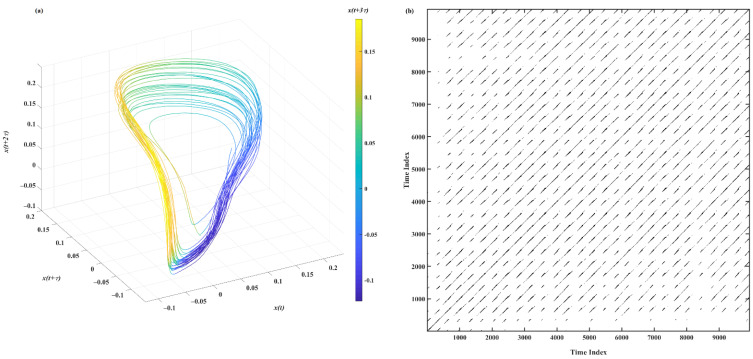
An example of (**a**) time-delay-reconstructed attractor and (**b**) the resulting RP obtained from one of the PPGs recorded in this study.

**Figure 8:**
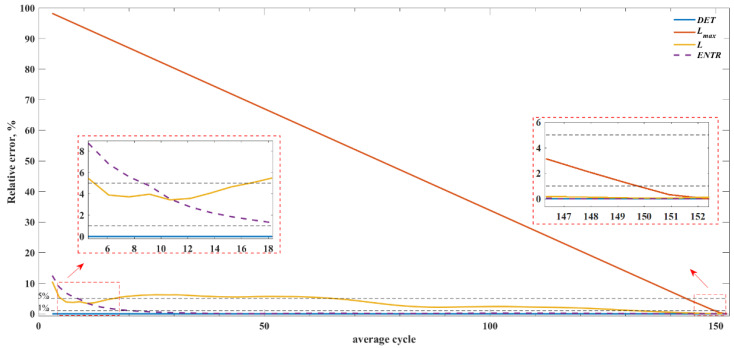
Relative error of the RQA features estimates as a function of the length of the time series.

Similarly to the Rössler system case described in Section 4.1, for each measured PPG time series, the time-delay-reconstructed attractor was computed. Then, for each reconstructed attractor, the set of RPs corresponding to the segment of the trajectory with length from 3 to 152 average cycles was calculated (i.e., 2.4 s to 122 s). The length of the average cycle was chosen, taking into account the physiological properties of the PPG signal. Specifically, the average cycle was set at 0.8 s—that is, the average duration of the cardiac cycle in an average healthy human subject [55]. Then, the RQA measures were estimated from the obtained RPs. An example of the time-delay-reconstructed attractor and the corresponding RP obtained from a 24.4 s-long PPG segment is shown in Figure 7. Figure 8 shows the average relative error of the RQA feature estimates as a function of the length of the time series. Table 3 summarizes the lower limits for the length of the time series, which provides RQA feature estimates with relative errors below 5% and 1% for time series with original and reduced sampling rates and reference RQA values for original time series.

## 5. Discussion

The main aim of this preliminary study was to evaluate the error associated with RQA feature estimates as a function of the length of the PPG time series and to identify recommended limits for the length of PPG recordings in order to keep the error below acceptable levels (e.g., 1%). In this study, PPG recordings measured in healthy individuals sitting comfortably in a relaxed position were used.

In addition, the relative error of RQA feature estimates was assessed for original and noisy time series generated using the chaotic Rössler model. The distributions of the relative error associated with the estimates of *DET*, *L_max_*, *L*, and *ENTR* shown in Figure 5a–d indicate specific patterns associated with the different RQA features. As seen in Figure 5a, accurate estimates of determinism can be achieved even for very short time series (e.g., 1500 points). The lowest acceptable length of the time series increases with increasing noise, as shown in Figure 5a and Figure 6 and Table 2, but *DET* remains the feature requiring the shortest time series length among the RQA features addressed in this study. It is of note that with high levels of added noise, the time series cannot be considered deterministic, as *DET* is significantly below 0.9 (Table 1).

The analysis of the error associated with the *L_max_* showed that accurate estimates of this feature—that is, the inverse of the divergence—cannot be achieved for short time series (Figure 5b and Figure 6), as *L_max_* requires long time series, i.e., longer than 48,500 points, for obtaining errors below 1%. It is of note that the average *L_max_* value for original data (i.e., 3763.9 points, Table 1) is significantly shorter than the used maximal time series length; nevertheless, the resulting lower limit for time series length is compatible with the maximal time series length used. Figure 5b and Figure 6 and Table 2 also clearly demonstrated that in the absence of noise, the lowest acceptable length of the time series is higher than that observed with noisy data due to the fact that the presence of noise significantly shortens the length of the maximal diagonal lines (Table 1).

The error patterns observed for *L* (Figure 5c), which is an important measure characterizing the average prediction time, show that the lowest acceptable length of the time series is longer for original data, and it decreases with increasing noise (in the range 0.065–0.15) due to the quick shortening of diagonal lines with noise induction (Table 1) while the determinism is preserved, and then it steadily increases again for higher noise. A similar pattern was observed for *ENTR* (Figure 5d and Figure 6), which quantifies time series complexity, (Figure 5d), suggesting that the observed trend attributed to high levels of noise disrupting data determinism (Table 1).

Overall, the results observed for the original and noisy Rössler model time series suggest that determinism can be accurately estimated even for short time series and that, if the time series is deterministic (i.e., if noise is sufficiently low), *L* and *ENTR* can be estimated accurately for substantially shorter time series than the reference length (i.e., 50,000). Specifically, *θ* < 0.2 time series with lengths above 25,000 points or 85 average cycles provide acceptable estimates if an error limit equal to 1% is set, and shorter time series could be used if higher errors are accepted (e.g., 10,000 points for 5% error limit).

The PPG signal inevitably contains a certain amount of measurement noise. The results shown in Figure 8 and Table 3 show that, similarly to the noisy Rössler model, *DET* can be correctly estimated even for very short lengths of PPG time series, specifically 1000 points (3.05 average cycles or 2.44 s). The *ENTR* can be evaluated with error below 1% for 7000-point (21.35 average cycles or 17.08 s) time series. For the estimates of *L* and *L_max_*, longer time series are required to reach error below 1%, specifically 43,000 (131.15 average cycles or 104.92 s) and 49,120 (149.75 average cycles or 119.8 s), respectively. However, the *L_max_* lower limit of the time series almost reaches the maximal time series length, and the actual *L_max_* value—unlike the results of the simulated data—is comparable with the maximal time series length, thus making it inapplicable for short recordings of the investigated PPG type.

The observed relationships between the error associated with each RQA feature estimate and the signal length, as reported in Figure 8, can be used to predict the average error level in practical applications in which only limited segments of the PPG are available. For example, if a 2.5 s PPG time series is available, accurate estimates of determinism can be expected, and the estimates of *ENTR* will be associated with an error equal to approximately 12%, whereas for 3.8 s PPG time series, the estimates of *ENTR* are associated with an average error lower than 5%; that may be a sufficiently low error in practical applications.

It is of note that the results presented in this study apply to the transmission-type NIR PPG signals collected in the reference environment; therefore, further studies are required to confirm limited-time-series-length-related error in dynamical property estimation using RQA for other types of PPG signals as well as for different experimental settings. Additionally, it is expected that these results apply to the PPGs with lower sampling rates, as seen in Table 3, where only minor changes in the time series length are observed for subsampled PPG data. However, the significantly different sampling rate of the signal may affect demonstrated results. Moreover, a more systematic investigation of the sampling rate effect in comparison with reference data with a high sampling rate and sufficient time series length is needed. Finally, an expansion of this study towards data whose trajectory is directly filtered in the state space might be beneficial for various applications; such noise reduction was reported to preserve nonlinear dynamical characteristics for heart rate variability data [56].

## 6. Conclusions

The results of this study can support a deeper understanding of the accuracy of the dynamical features estimated from experimental PPG time series of limited length in real-world applications—for example, health monitoring using wearable devices. Specifically, this study investigated the effect of the length of the time series on the accuracy of the estimates of the RQA features estimated from NIR light-based PPG signals. As a result, minimum requirements for the length of the PPG time series needed to obtain sufficiently accurate estimates of the dynamical properties of the signal could be defined as relevant to practical studies and experiment design.

## Figures and Tables

**Table 1:** Values of determinism, maximal diagonal line length, average diagonal line length, and entropy obtained for long Rössler’s *x* time series for three different noise levels.

Noise Level, *θ*	*DET*	*L_max_*	*L*	*ENTR*
0	0.9992	3763.9	60.4329	4.6079
0.245	0.4138	7.52	2.3379	0.7544
0.5	0.0910	3.24	2.0455	0.1765

**Table 2:** Time series length corresponding to relative errors equal to 5% and 1% in the estimates of determinism, maximal diagonal line length, average diagonal line length, and entropy for three different noise levels of the Rössler time series. The values highlighted indicate the lower limit of the length of the time series required for accurately estimating the four features.

Noise Level, *θ*	*E_l_*	*DET*	*L_max_*	*L*	*ENTR*
0	5%	1500	46,000	4000	10,500
	1%	1500	48,500	16,000	26,000
0.245	5%	3000	36,500	2500	7500
	1%	17,000	46,000	3500	30,000
0.5	5%	5000	41,500	11,000	42,000
	1%	7000	47,000	13,500	43,500

**Table 3:** Average values of the length of the time series corresponding to relative error below 5% and 1% and the reference RQA values.

	Lower Time Series Length Limit, Average Cycles						Reference RQA Values (409.6 Hz)
	409.6 Hz		204.8 Hz		102.4 Hz		
	*E_l_*, 5%	*E_l_*, 1%	*E_l_*, 5%	*E_l_*, 1%	*E_l_*, 5%	*E_l_*, 1%	
*DET*	3.05	3.05	3.05	3.05	3.05	3.05	0.998
*L_max_*	144.82	149.39	143.38	150.28	143.39	150.25	49,859
*L*	65.55	131.10	64.19	132.79	64.02	133.02	144.38
*ENTR*	7.62	21.34	9.65	22.05	10.67	24.39	5.73

## Data Availability

Not applicable.
